# Pancreatic Cancer

**DOI:** 10.1155/2015/809036

**Published:** 2015-03-24

**Authors:** Niccola Funel, Marco Del Chiaro, Djuna L. Cahen, Johanna Laukkarinen

**Affiliations:** ^1^Department of Translational Research and New Technologies in Medicine and Surgery, University of Pisa, 56124 Pisa, Italy; ^2^Pancreatic Surgery Unit, Division of Surgery, Department of Clinical Science, Intervention and Technology (CLINTEC), Karolinska Institutet, Center for Digestive Diseases, Karolinska University Hospital, K53, 14186 Stockholm, Sweden; ^3^Department of Gastroenterology and Hepatology, Erasmus University Medical Center's, Gravendijkwal 230, P.O. Box 2040, 3000 CA Rotterdam, Netherlands; ^4^Department of Gastroenterology and Alimentary Tract Surgery, Tampere University Hospital, Teiskontie 35, P.O. Box 2000, 33521 Tampere, Finland

Pancreatic tumors are challenging diseases. Pancreatic adenocarcinoma (PDAC), in particular, is associated with significant morbidity and mortality. Another group of exocrine tumors, intraductal papillary mucinous neoplasia (IPMNs), represents the paradigm of progression of malignancy and their connection and differentiation with respect to pancreatic ductal adenocarcinoma (PDAC) which is still complicated. In this special issue, we would like to offer readers an overview of some important aspects for the two most representative exocrine tumors as IPMN and PDAC. The original communications within this special issue fall into four different categories: pancreatic surgery, management of IPMN patients, treatment of pancreatic tumors, and, last but not least, some important aspects of basic science in PDAC.

*Surgery*. Curative resection is considered the only potential for cure in pancreatic cancer. Complete macroscopic tumor resection is perhaps the most relevant predictor of long-term survival in PDAC. Locally advanced pancreatic tumors can involve vascular structures and adjacent organs, and thus vein resections and even resections of additional organs may be needed to achieve the goal. Improving surgical methods to achieve this goal is crucial in the treatment of this disease.

In pancreatic surgery, postoperative pancreatic fistula (POPF) remains the most challenging complication after resections, whether pancreatoduodenectomy or distal. POPFs are contributing significantly to prolonged hospitalization and mortality. Several studies using various anastomotic and sealing techniques have been studied to reduce the amount of POPFs. Some previous investigations have shown that pancreatic trauma and the following inflammation are preceding postoperative complications and they should be avoided. Patients with normal, acinar-cell rich pancreas are in higher risk to develop POPF. One promising technique for pancreatoduodenectomy is the Finnish binding pancreaticojejunal anastomosis (FBPJ), where the pancreatic trauma is minimized by avoiding sutures running through the pancreatic tissue. The preliminary previous studies with this technique have shown reduced amount of POPF after pancreatoduodenectomy.

*IPMN Management*. Intraductal papillary mucinous neoplasms of the pancreas (IPMNs) are a high prevalence neoplasm of the pancreas. IPMNs can progress from adenoma to invasive cancer through a process very similar to the one of the colonic polyps. At the moment the management of pancreatic IPMN is suggested by the Guidelines of the International Association of Pancreatology and by the European Guidelines for Cystic Tumors of the Pancreas. However, till now, the available evidence about the diagnosis and treatment of those tumors is quite low and the clinical decisions are made on the basis of expert consensus (more than guidelines) and local expertise. Even though IPMNs represent in one side an opportunity to prevent pancreas cancer (through an early detection and treatment of precancerous lesions), considering the high risk associated with pancreatic surgery, there is a potential risk to overtreat patients that might never develop cancer. Even the decision making in the intraoperative management of IPMNs is complicated: the extent of resection, the role of parenchyma sparing procedure, and the role and significance of margins analysis are argument under investigations and have not been yet defined.

To make the overall picture even more complicated, the accuracy of the current imaging modalities is reported very low even in high volume centers. In a recent series the overall accuracy in defining preoperative diagnosis in cystic tumors of the pancreas was inferior to 70% and even the use of endoscopic ultrasound plus FNA was not able to increase the results. More large studies are needed in order to better understand the natural history of these tumors, to discriminate the ones that can progress to cancer from the ones with low aggressive behavior. At the same time, new insight into the field of diagnostic is necessary in order to increase the accuracy of imaging modalities. For the reasons mentioned above and for the tremendously high prevalence, IPMNs represent today maybe the most challenging area of interest in pancreatology, a great opportunity to reduce the pancreas cancer mortality, but also one of the most dangerous clinical areas.

*Pancreatic Cancer Treatments*. Pancreatic adenocarcinoma is one of the most deadly cancers, with an overall 5-year survival of 5%. Complete surgical resection provides the only chance for cure. Unfortunately, most patients are diagnosed with locally advanced or metastatic disease. Chemotherapy, with and without radiation, has been investigated in both neoadjuvant and postoperative settings. At present, multimodality therapy seems to be the future direction. However, the sequence of surgery, chemotherapy, and radiation remains to be determined. In the review of this special issue, the authors examined available data on neoadjuvant treatment in resectable patients and in patients with borderline resectable or locally advanced disease.

Of course, the need for new therapies is undisputed. One of the latest local treatments is high-intensity focused ultrasound (HIFU), a noninvasive and safe technique to ablate solid tumors. In this special issue, the authors have reviewed all 3022 cases, described in the literature, with respect to safety and efficacy.

*Basic Science of PDAC*. In the papers published in this special issue, the authors treated three molecular aspects regarding the PDAC: indicator tryptase, ATP-binding cassette (ABC) transporters, and micro-RNA (miRNA) expression. These molecules are involved in the progression, chemoresistance, and survival of PDAC patients, respectively. In particular, M. Ammendola et al. have shown the role of tryptase in PDAC which is associated with mast cells to increase the microvascular density (MVD) in tissue PDAC. This could play an important role in vascularization, the absorption of drugs, and chemoresistance of PDAC. In fact, drug chemoresistance of PDAC cells is recognized as the primary cause of failure of chemotherapy. Although biochemical behaviors, including low drug concentration in the tumor, may contribute to clinical resistance, different types of molecules, including ATP-binding cassette transporters, are the main actors in the extrusion of drugs in different tumors. In particular, some single nucleotide polymorphisms (SNPs) of the three most important family genes of ABC (ABCB1, ABCC1, and ABCG2) are associated with a lower risk of developing pancreatic cancer and an increased sensitivity to gemcitabine than other haplotypes. Finally, the most important epigenetic factors, such as miRNAs, were confirmed to be “micromolecules,” regulating “the great effects.” Their deregulation modulates several important processes in PDAC, including differentiation, tumor progression, and epithelial-mesenchymal transition (EMT). Nevertheless, miRNAs affect overall survival and chemoresistance of PDAC patients. Aberrant expression of three most important miRNAs (miR-21, miR-155, and miR-101) is strongly associated with PDAC, IPMN, and nonmalignant lesions, respectively.

*Niccola Funel* *Niccola Funel* *Marco Del Chiaro* *Marco Del Chiaro* *Djuna L. Cahen* *Djuna L. Cahen* *Johanna Laukkarinen* *Johanna Laukkarinen*

